# Utilizing Lipid Bond Technology With Molecular Lipid Complex to Provide Lipid Treatment for Damaged Hair

**DOI:** 10.1155/drp/5385312

**Published:** 2025-08-07

**Authors:** Nam Hai Lai, Thi Hong Ngoc Dang, Thu Thuy Nguyen, Duong Thuc Quyen Phan

**Affiliations:** Department of Research and Innovation, Moonshot Biotech PTE. LTD., 9 Raffles Place, #29-05, Republic Plaza, Singapore 048619

**Keywords:** damaged hair, delivery/vectorization/penetration, emulsions, hair treatment, Lipid Bond

## Abstract

Human hair fibers are mainly composed of proteins, lipids, and water. In particular, lipids play an important role in keeping hair healthy, stabilizing its structure, affecting shine, feel, manageability, and strength. In addition to each person's physical condition and constitution, the cause of reduction and loss of hair lipids also comes from external causes such as UV, pollution, and specially styling chemicals. A decrease in hair lipid content correlates with reduced tensile strength, diminished shine, increased breakage, and hair damage. In this study, we focus on Lipid Bond Technology with small molecule real lipids derived from plant oil triglycerides, known as 369LAB Lipid Bond, reverse chemical and environmental damage by restoring lipid bonds in hair, regenerates hair structure, and improves hair strength. Research results show that 369LAB Lipid Bond has an average particle size of 39.83 nm, helping to penetrate deeply into the hair; replace lost lipids, restore lipids to damaged hair. After one use, the total amount of lipid restored is equivalent to natural hair before damage. It is nonsticky and does not clog hair follicles. SEM images show morphological improvement in the integrity of the epidermis and regeneration of lipid layers immediately, with lasting repair even after discontinuation of use. The amount of force that breaks individual hair strands in testing shows that 369LAB Lipid Bond makes hair stronger. The Lipid Bond Technology not only strengthens hair while using the product but also maintains its healthy resilience long after. The current research will provide the breakthrough for new applications in cosmetic, skin, and hair care products, to address the remaining difficulties and challenges in the treatment of damaged hair.

## 1. Introduction

**Figure 1:**
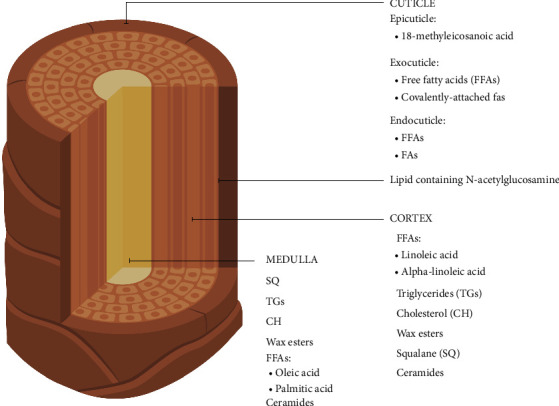
Hair transverse layers and lipid composition.

Lipids are thought to play an important role in all compartments of hair and skin substructure [1] and also in involvement of hair development and function [2, 3]. The lipid in hair is not only crucial for maintaining its structural integrity and physical properties but also varies in concentration across different layers of the hair shaft, underscoring its multifaceted role in hair biology and functionality [4, 5]. The hair shaft, a filament-like structure originating from the epidermis through the hair follicle, is a complex biological material composed of three concentric layers (Figure 1) [6]. The outermost layer, known as the cuticle, consists of planar cells that overlap like shingles on a roof. This cuticular layer forms a protective barrier strengthened by a lipid network, which constitutes only a thin layer of the hair's bulk but serves a critical role in maintaining the hair's ability to interact with water and shielding it from environmental stresses [7]. The epicuticle comprises a hydrophobic lipid monolayer of 18-methyleicosanoic acid (18-MEA), also present in the β-layer of the cell membrane complex (CMC) that keeps the hair cells attached [8–11]. The lower level of the cuticle contains covalently bound fatty acids and free fatty acids (FFA) [12]. The cortex comprises the major keratinized part of the hair and consists of elongated, closely packed cells parallel to the fiber axis, with a very low lipid content. However, oxidative metabolites derived from integral fatty acids, such as linoleic and alpha-linoleic acids, are noncovalently lipids and bind to melanin granules [13]. The medulla, which is the innermost portion of the terminal hair shaft, consists of a loose layer of medullary cells and empty vacuoles, which are significantly stabilized by structural lipids [14, 15]. Recent studies have confirmed the presence of lipids in this region, with concentrations found to be significantly higher—between 3 and 20 times more—than in the surrounding cortex [7]. Recent lipid profile results indicate that the medulla comprises squalene, fatty acids such as oleic acid, wax esters, and FFAs such as palmitic acid [16, 17]. Another recent study confirms the high lipid concentration containing a mixture of nonesterified and esterified lipids and carboxylate soaps in various proportions [18].

In the hair, a laminated structure of lipid molecules such as fatty acids, ceramides, glycolipids, and cholesterols plays a role in the protection [6]. This lipid barrier is essential for preventing the penetration of foreign matter and the loss of internal moisture [6, 19, 20]. Commonly used chemical treatments, such as bleaching, straightening, and coloring, profoundly alter the cortex's structure upon reaching it by breaking covalent bonds, oxidizing lipids, and degrading keratin and other associated proteins [21–25]. Bleaching removes the uppermost layer of 18-MEA and free lipids, deteriorating the cuticle and leading to a hydrophilic surface with increased friction. The absence of 18-MEA and degradation of the epicuticle results in hair that feels “dry,” brittle, messy, and difficult to comb. More than 80% of 18-MEA is removed in a single bleaching treatment, as the bleaching process oxidizes the cysteine bonds to cysteic acid [26, 27]. The entry of external molecules is essential to mitigate the damage caused to the structure by neutralizing the generated charged species, restoring hydrophobicity, and partially rescuing mechanical properties [28–30].

The use of plant oils in hair care has grown significantly in recent years. Furthermore, plant oils are recognized for offering hair benefits that go beyond the conventional roles of lubrication and enhancing shine. Many plant oils contain essential fatty acids, vitamins, and antioxidants that can strengthen hair, improve moisture retention, and protect against environmental damage. In the study of the effects of plant oils on hair, oils like argan, coconut, and jojoba oil are rich in nutrients that can help repair damaged hair, reduce frizz, and enhance hair elasticity [31–34]. Studies have shown that the interaction between different oils and hair is primarily influenced by the affinity of the molecules for the CMC, which acts as an adhesive between the cells that bind the cuticle and cortex together, as well as by the molecular structure and size [31–38]. The topic of plant oil absorption into hair remains controversial, with perspectives based on scientific studies and practical observations. Molecular size and oil polarity play a crucial role in absorption [34]. Oils with smaller molecular structures, such as coconut oil, due to their fatty acid composition (particularly lauric acid), can penetrate the hair and interact with hair proteins [35, 37]. In contrast, unsaturated oils like argan oil tend to stay on the hair's surface because of their bulkier molecular structure [39]. Debates over solubility and permeability are among the reasons why the application of plant-based triglycerides in the cosmetic industry faces many challenges.

Considering the aforementioned data, the objective of the present study was to utilizing lipid bond technology with molecular lipid complex to provide lipid treatment for damaged hair. The Lipid Bond Technology of MOONSHOT BIOTECH PTE. LTD. is completely a new technology, registered for patent in the US—“Process for production of nano-microemulsion system of plant oil triglycerides”—US20200346174A1 [40]. The production of 369LAB Lipid Bond microemulsions of triglycerides allows the formation of small droplets that imitate the biological hair lipid composition of uniform size, which are soluble and have long-term stability in water without changing the activity and structure. This property, therefore, helps to increase the using efficiency, in particular increasing the absorption and bio availability, that can be applied on an industrial scale. In addition, small molecule real lipids also demonstrate antioxidant properties, reduce ROS index, and protect cells from the effects of UV rays [41]. This study aims to evaluate the effectiveness of reversing hair damage by restoring lipid bonds in hair, rebuilding hair structure and improving hair strength, with 369LAB Lipid Bond material created by Lipid Bond Technology.

## 2. Materials and Methods

### 2.1. Materials

The 369LAB Lipid Bond ingredient was researched and developed by MOONSHOT BIOTECH PTE. LTD and created through a patented process in the United States—“Process for production of nano-microemulsion system of plant oil triglycerides”—US20200346174A1 [40].

All chemicals, solvents, and standards used in the present study were purchased from Merck and Sigma–Aldrich.

### 2.2. Equipment

The Zetasizer Ultra (Malvern, United Kingdom) was employed to measure particle and molecular size, particle charge, and particle concentration. A vacuum pump equipped with a Whatman GF/A filter (Whatman Plc, Maidstone, UK) was utilized to remove particulate matter. The surface morphology of the hair fibers was imaged using a field emission scanning electron microscope (FE-SEM, S4800, Hitachi, Japan). Hair tensile strength was evaluated using a Force Gauge (Sauter FK250, China).

### 2.3. Hair Samples

Natural black Asia hairs were used in the current study. The hair samples had never been exposed to chemical treatment, such as perming, dyeing, and/or bleaching. The samples were sealed in zip-lock bags and stored in a dark place, in the laboratory at a temperature of 23°C–25°C and a relative humidity of 50%–60%.

### 2.4. Preparation of Artificially Damaged Hairs

Different bleached hair tresses, with or without the evaluated products, were used to assess their protective effect during the bleaching process. Hair bleaching mixture is mixed from bleaching powder (ammonium persulfate) and oxidizing agent (12% H_2_O_2_ solution) in a 1:1 ratio.

The hairs were divided into approximately 2 g, 15 cm lengths for each tress, and secured at the nontip end. The hair was bleached 2 times. For the first time, 16 g of the bleaching mixture was applied to the hair at room temperature. The mixtures were evenly applied to the hair tresses, which were then wrapped in aluminum foil and left for 45 min. Then, the hair sample was rinsed with tap water for 1 minute and then dried with towel. The second bleaching was carried out using 16 g of the bleaching mixture for 15 min. After that, the hair sample was washed with warm water, and then dried with towel and air-dried. The presented artificially damaged hairs were used in the following experiments.

### 2.5. Treatment of Damaged Hair

The bleached hair tresses of 400 mg was submerged in 10 g 369LAB Lipid Bond (70% wt of 369LAB Lipid Bond and 30% wt water) for 15 min. The hair strands were massaged during this process. After that, the bleached hair sample was rinsed with water and dried.

### 2.6. Determined the Total Hair Lipid Content

Total lipid was extracted according to the method of Bligh and Dyer [42]. In brief, 400 mg hair sample was extracted with 6 mL mixture of methanol: chloroform (2:1, v/v) in glass tubes with 20 mL Teflon-lined screw caps (3 replicates). The samples were then heated at 50°C for 1 h followed by filtration to remove particulate matter using a vacuum pump through a Whatman GF/A filter (Whatman Plc, Maid-stone, UK). The residue was re-extracted twice. Chloroform (4 mL) and distilled water (4 mL) were added to the combined filtrate and the organic phase was separated. After drying over Na_2_SO_4_, the solution was filtered and evaporated under reduced pressure to obtain the total lipid which was stored in chloroform at −5°C.

### 2.7. SEMs Analysis

SEMs use a focused beam of electrons to produce images of objects that have been magnified up to 5000 times, revealing detail and complexity inaccessible with light microscopy. The study used the FE-SEM S4800 (Hitachi, Japan) with the accelerating voltage of 10 kV to take images of the hair fiber surface and the hair cross-section before and after treatment with 369LAB Lipid Bond. Samples used are Natural black Asia hair, bleached hair, and bleached hair after treatment with 369LAB Lipid Bond.

### 2.8. Hair Strength Test

The sample hairs of 200 mg each were prepared. All tresses were washed daily with shampoo base formulation (Table 1) for 8 minutes and then rinsed with water, and then dried with towel and air-dried. All hair tresses are described below:

- Group 1: Bleached hair treated with control shampoo.
- Group 2: Bleached hair treated with control shampoo + 369LAB Lipid Bond.

Tensile strength of hair samples was measured using a Force Gauge (Sauter FK250, China). The instrument exerts a constant speed of extension of 25 mm/sec on a single hair fiber and extends the fiber until it breaks. Break stress (break load) was measured using average values of 1 hairs/sample. All measurements were made in an air-conditioned laboratory to guarantee a constant room temperature of 23°C–25°C and 50%–60% relative humidity.

The results obtained above represent the combing force, in Newtons, required to break the hair fiber. The mean from each set of breakage measurements is determined for swatches.

### 2.9. Statistical Analysis

Statistical analysis was conducted using SPSS 25.0 Software. A one-way ANOVA followed by Tukey's test was used for lipid content comparisons, and an independent-samples *t*-test was applied for hair strength testing. Statistical significance was determined as *p* < 0.05, *p* < 0.005 was considered highly significant.

## 3. Results and Discussion

### 3.1. Zetasizer Ultra Particle Size Analyzer

**Figure 2:**
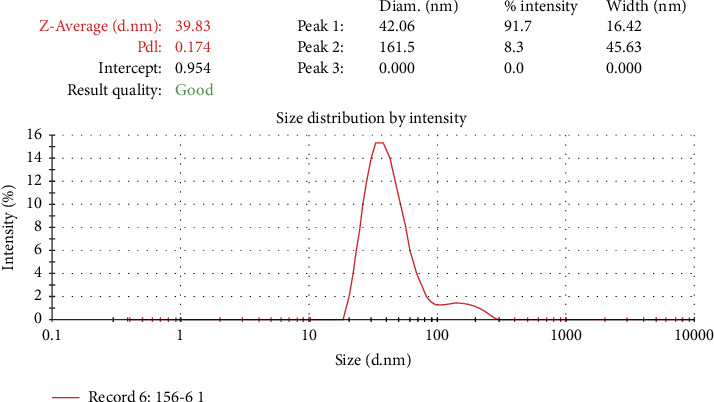
Size distribution of 369LAB Lipid Bond.

Particle size analysis showed the presence of small particles size with a polydispersity index (PDI) value of 0.174 with an intercept of 0.954, ranging in size from 20 to 300 nm. Thereby, the 369LAB Lipid Bond has a small size (Figure 2), with the average particle size (Z-Average) found to be 39.83 nm.

Su et al. [43] tested nanoemulsions with 3 sizes (80-200–500 nm) to demonstrate their ability to penetrate into intact hair follicles. The results indicated that the nanoemulsion was absorbed by antigen-presenting cells residing in the keratinocytes and peri-follicular sites. They finally concluded that 80 nm nanoemulsions could penetrate the viable cuticle as well as fill the entire hair follicle, while 500 nm nanoemulsions could not effectively penetrate the cuticle and only migrated transfer along the hair follicle and finally the 200 nm sized nanoemulsion shows a moderate axial distribution effect between the three sized nanoemulsion.

In another study, Patzelt et al. [44] found that the optimal size for penetrating hair follicles ranges from 400 to 700 nm, each size capable of selectively targeting different structures within the follicle hair. Particles with a size of 470 nm are said to have the deepest penetration ability into the bulge area of the hair follicle.

Rahmasari et al. [45] demonstrated in their tests that the nano concentration of argan oil is directly proportional to hair growth activity. Thanks to being rich in ingredients such as vitamin E and oleic acid, it helps provide oxygen and nutrients to hair cells, especially hair follicles. Olive oil has good penetration-enhancing properties for topical drug delivery (especially its oleic acid content of between 70% and 85% [46]).

Based on the aforementioned studies, lipid particles derived from triglycerides, with a size smaller than 500 nm, are thought to effectively and rapidly penetrate deep into the hair follicles and shaft, offering long-lasting treatment and care for damaged hair.

### 3.2. Total Hair Lipid Content

**Figure 3:**
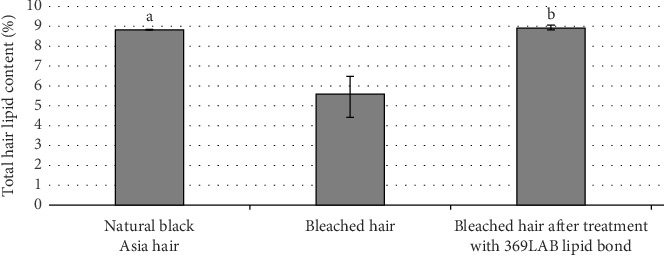
Total lipid content of the study samples. ^a,b^Indicates significant difference *p* < 0.005.

The total lipid in natural hair is 8.703%. After bleaching, the total lipid content decreased to 5.506%; after just 1 use is restored to equal to the index of natural hair (8.811%) (Table 2, Figure 3).

It can be observed that a significant amount of lipids in the hair is lost after the bleaching process (Figure 3). This is due to unwanted oxidative damage across the entire hair fiber. The penetration of peroxide may be facilitated by the substantial structural degradation caused to the hair's cuticle layers. Studies have shown that bleaching agents composed of alkaline peroxide-persulfate can attack the thioester groups that link 18-methyl eicosanoic acid to surface proteins. This reaction partially removes the hydrophobic (lipid) barrier and generates sulfuric acid (mainly sulfonate groups) on and within the hair fiber's surface [47–50]. Free radical reaction leading to lipid breakdown and further protein degradation, not only at disulfide bonds but also at peptide bonds [51]. As a result, a large amount of lipids on and within the hair fiber is lost, severely impacting hair structure.

The use of 369LAB Lipid Bond treatment has helped restore a significant portion of the total lipid content in the hair structure. The results showed that there was no statistically significant difference between the natural hair and the bleached hair treated with 369LAB Lipid Bond, indicating that 369LAB Lipid Bond was able to restore lipid content to a level equivalent to undamaged hair (Figure 3). Marsh et al. [52] demonstrated that the presence of external materials within the fiber can interfere with molecular interactions in the cortex. After penetrating through the intact CMC of virgin hair, triglyceride oils have the ability to establish hydrophobic interactions with keratin chains and matrix proteins. These interactions create competition for both intermolecular and intramolecular interactions within the matrix and keratin chains [39].

With its small particle size, nongreasy nature, and no clogging of hair follicles, 369LAB Lipid Bond deeply penetrates the hair. It replaces lost lipids, restores lipids to damaged hair, and effectively maintains the total lipid content after treatment.

### 3.3. SEMs Analysis

To observe the morphological changes on the hair surface before and after treatment with 369LAB Lipid Bond, the study utilized the FE-SEM S4800 (Hitachi, Japan) at 10 kV, taken at magnifications of 2000 to 5,000, yielding the following results.

**Figure 4:**
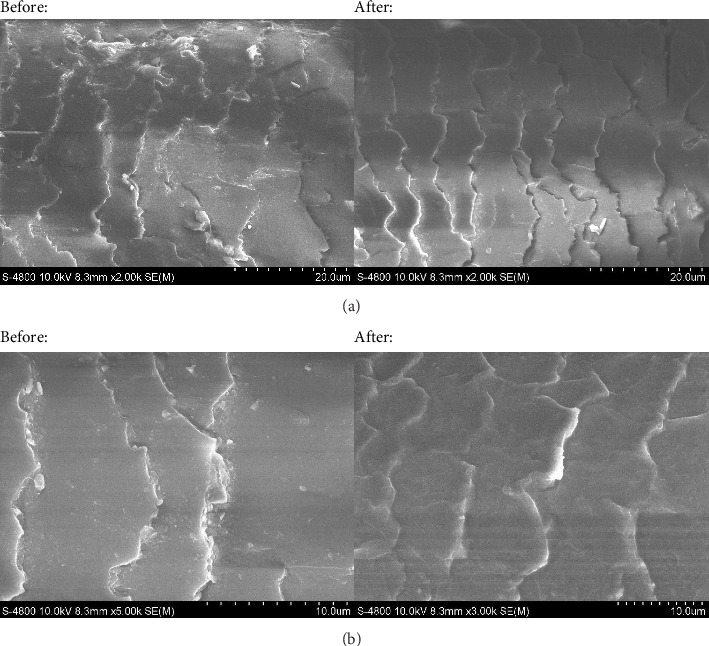
SEM images of the hair fiber surface before and after treatment with 369LAB Lipid Bond. (a) Natural black Asia hair; (b) bleached hair.

Observation shows that the cuticle of damaged hair is lifted (Figure 4(a)). Several pits and bond breakages are easily visible. After treatment with 369LAB Lipid Bond, the level of surface damage is significantly reduced, and the hair cuticles appear relatively intact and uniform. The hair cuticle was severely damaged after bleaching (Figure 4(b)). The peeling of the cuticle is due to ammonium persulfate, a strong oxidizing agent that helps open the hair cuticle, allowing hydrogen peroxide to penetrate deeper [53]. This opening of the cuticle increases hair porosity, making the hair more prone to moisture loss and environmental damage. After treatment with 369LAB Lipid Bond, the level of cuticle damage was significantly reduced, as clearly observed in the SEM images.

To gain a deeper insight into the morphological changes within the hair strand before and after treatment with 369LAB Lipid Bond, the study proceeded with the use of the FE-SEM S4800 (Hitachi, Japan), yielding the following detailed results.

**Figure 5:**
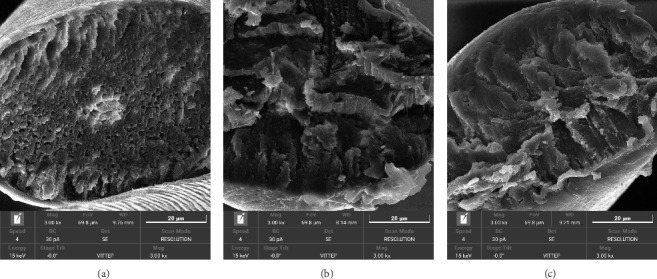
SEM images of the morphological changes within the hair strand before and after treatment with 369LAB Lipid Bond. (a) Natural black Asia hair; (b) bleached hair; (c) bleached hair after treatment with 369LAB Lipid Bond.

In normal hair (Figure 5(a)), the morphological layers exhibit a uniform structure with no visible signs of damage. It is indicated that the disulfide bonds, peptide bonds, and other molecular interactions maintain the integrity of the layers, creating a cohesive and intact hair structure [54]. However, following the bleaching process, a considerable loss of lipids is observed (Figure 5(b)), likely a result of oxidative damage occurring throughout the hair shaft. Research on the hair bleaching process has demonstrated the breakdown of the cuticle layers facilitates deeper penetration of peroxide, leading to significant structural degradation [53]. As a result, the internal layers of the bleached hair show clear signs of damage, including pitting and bond breakage. Upon treatment with 369LAB Lipid Bond, the extent of structural damage is markedly reduced (Figure 5(c)). SEM imaging reveals a restoration of the internal hair layers to a more uniform and completes state.

The 369LAB Lipid Bond penetrates deep into the hair to reduce the swelling tendency of the cuticle, thereby limiting the upward curvature of the surface cuticle. This minimizes epidermal damage and reduces protein loss. The oil molecules that penetrate the hair have a high affinity for the protein, filling the gaps in the hair's protein structure and, as a result, helping to minimize the loss of melanin pigmentation.

### 3.4. Hair Strength Test

**Figure 6:**
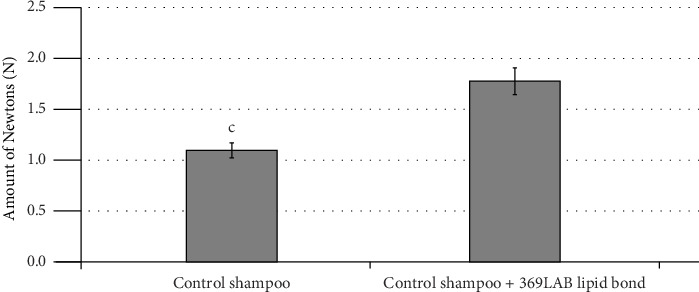
The amount of force to break each strand in hair strength in test. ^c^Indicates significant difference *p* < 0.005.

The obtained results in the tensile test showed that the use of the formulations with 369LAB Lipid Bond promoted an increase of the hair fiber strength, which was not observed in the hair fiber with the application of the control shampoo (Figure 6).

The formulation containing 369LAB Lipid Bond demonstrated a significantly greater improvement in the mechanical properties of the hair fiber compared to the control. Since this analysis focuses on the internal structure of the hair, particularly the strength of the cortex, it can be inferred that the components of triglyceride played a pivotal role in treating the internal regions of the hair fiber, leading to enhanced structural integrity [34, 37, 39].

In summary, the formulations enriched with the 369LAB Lipid Bond effectively improved the condition of the hair cuticles, resulting in increased softness, hydration, and tensile strength. These improvements were not observed with the control shampoo. Thus, 369LAB Lipid Bond represents a promising ingredient for inclusion in hair care formulations, offering potential benefits in enhancing hair gloss, softness, and repairing damage.

## 4. Conclusions

Research results show that 369LAB Lipid Bond has a small size from 20 to 300 nm to help penetrate deeply into hair; replace lost lipids, and restore lipids for damaged hair; after 1 use, the total lipid is restored equal to the natural hair before damage. It is not sticky and does not clog hair follicles. SEM images show the morphological improvement in cuticle integrity, internal hair layers and rebuilding of the lipid layers after one time. In addition, the amount of force to break each strand in hair strength test shows 369LAB Lipid Bond makes hair stronger (higher tensile strength).

Research shows that Lipid Bond Technology creates small molecule real lipids that imitate the biological lipid composition of hair. This restores lipid loss due to time or color treatment, and chemical treatment and thus restores the health of the hair. This technology reconstructs and revitalizes lipid bonds deep within the hair. 369LAB Lipid Bond is both safe and sustainable, resolving the issue without causing stickiness, ensuring long-term effectiveness. The Lipid Bond Technology's internal repair not only strengthens hair during product use but also preserves its healthy resilience even afterward. This foundational benefit opens up new possibilities for applying Lipid Bond Technology and 369LAB Lipid Bond ingredient across cosmetics, skincare, and haircare products.

## Figures and Tables

**Table 1:** Shampoo base formulation.

Ingredients	Control shampoo % (w/w)	Control shampoo + 369LAB lipid bond % (w/w)
Aqua (water)	q.s. to 100%	q.s. to 100%
369LAB lipid bond	0.00	3.00
Tetrasodium EDTA	0.10	0.10
Sodium C14-16 alpha olefin sulfonate	4.00	4.00
Cocamidopropyl betaine	3.00	3.00
Disodium laureth sulfosuccinate	10.40	10.40
Stearyl alcohol, cetyl alcohol	3.00	3.00
Citric acid	1.00	1.00
Phenoxyethanol and ethylhexylglycerin	0.80	0.80

**Table 2:** Total lipid content of the study samples.

Samples	No.	Fresh sample (g)	Total lipid (g)	% of total lipid
Natural hair	1	0.2278	0.0198	8.692
	2	0.2331	0.0206	8.837
	3	0.3007	0.0258	8.580

Bleached hair	1	0.2848	0.0189	6.636
	2	0.2042	0.0089	4.358
	3	0.4164	0.0230	5.524

Bleached hair after treatment with 369LAB Lipid Bond	1	0.2224	0.0199	8.948
	2	0.3124	0.0284	9.091
	3	0.6291	0.0528	8.393

## Data Availability

The raw data that support the findings of this study are available from the corresponding author upon reasonable request.
